# Prevalence of poor self-rated health and common mental disorder among persistently precarious employed adults

**DOI:** 10.1093/occmed/kqaf064

**Published:** 2025-09-22

**Authors:** Andrew Pulford, Michael Green, Daniel Kopasker, Alastair Leyland, Srinivasa Vittal Katikireddi

**Affiliations:** MRC/CSO Social and Public Health Sciences Unit, University of Glasgow, Glasgow, G12 8TB, Scotland, UK; Public Health Science, Public Health Scotland, Gyle Square, 1 South Gyle Crescent, Edinburgh, EH12 9EB, Scotland, UK; MRC/CSO Social and Public Health Sciences Unit, University of Glasgow, Glasgow, G12 8TB, Scotland, UK; Department of Obstetrics and Gynecology, Duke University Medical School, Durham, NC 27513, USA; MRC/CSO Social and Public Health Sciences Unit, University of Glasgow, Glasgow, G12 8TB, Scotland, UK; MRC/CSO Social and Public Health Sciences Unit, University of Glasgow, Glasgow, G12 8TB, Scotland, UK; MRC/CSO Social and Public Health Sciences Unit, University of Glasgow, Glasgow, G12 8TB, Scotland, UK

## Abstract

**Background:**

The aftermath of the 2008 recession created conditions in which precarious employment may have become more common in the UK. We characterized two distinct periods: high unemployment/lower income (2011–5) and lower unemployment/stagnant income (2015–9).

**Aims:**

We aimed to describe the prevalence and persistence of these dimensions of precarious employment over two time periods (2011–5 and 2015–9) and investigate the prevalence of precarious employment patterns, poor self-rated health and common mental disorder.

**Methods:**

We used the UK Longitudinal Household Study to create employment history sequences for three dimensions of precarious employment using two four-wave samples. We applied latent class analysis to sequenced data to identify people persistently exposed to each dimension of precarious employment. We calculated age-sex standardized prevalence of poor self-rated health and common mental disorder by latent class for each precarious employment dimension.

**Results:**

Our analysis included 16 161 (2011–15) and 12 963 (2015–19) individuals. We found a latent class representing persistent exposure to each of the three precarity dimensions, but little overlap between these classes. Latent classes were very similar for both study periods. Prevalence of poor self-rated health and common mental disorder increased for most latent classes between 2011–15 and 2015–19. Employment discontinuity had the highest prevalence out of the precariously employed latent classes for both outcomes.

**Conclusions:**

A minority of workers in the UK were in a state of chronic precarity, which did not change substantially between the two time periods. Experiencing persistent employment discontinuity was the dimension of precarity with the highest levels of poor health.

Key learning points **What is already known on this topic**

- The aftermath of the 2008 recession in the UK created economic conditions in which precarious employment may have become more common; up to 2015, this was characterized by high unemployment and lower income; from 2015 to 2019, this was characterized by lower unemployment but stagnant income.
- A previous systematic review demonstrated that persistent precarious employment is associated with negative health outcomes, including self-rated health and common mental health problems.
- However, exploration of different dimensions of persistent precarity has been limited in terms of the prevalence of both exposure to these specific dimensions and health outcomes.

**What this study adds**

- Our findings indicate a minority of workers in the UK who are in a state of chronic precarity, which did not change substantially between the two time periods we investigated.
- Our study highlights little overlap between groups of working-age adults in the UK persistently exposed to these dimensions of precarious employment (non-permanent employment, employment discontinuity and multiple employment); the prevalence of poor self-rated health and common mental disorders increased for most latent classes.
- Of the dimensions of precarious employment, people experiencing persistent discontinuity had highest prevalence of poor self-rated health and common mental disorder; prevalence of common mental disorder among the employment discontinuity class was 19% in 2011–15 and 20% in 2015–19, compared with 13% and 14% for the continuous employment class.

**What impact this may have on practice or policy**

- Poor self-rated health and common mental disorders became more prevalent for most employment history latent classes; however, adults experiencing persistent employment discontinuity should be considered as a priority group for public health intervention.
- Due to a lack of overlap between precarity dimensions, dimension-specific policy interventions may be necessary responses to persistent precarious employment, but do little to aid those experiencing precarity in other dimensions.
- Further research using robust quantitative methods is required to better understand the extent to which the relationship between dimensions of precarious employment and health outcomes is causal.

## INTRODUCTION

Precarious employment is a multi-dimensional concept including contractual insecurity, income insecurity, lack of rights, limited progression opportunities, and imbalanced power relations [1–3]. It is an important health determinant [1], with risks to mental health potentially comparable unemployment [4].

Drivers of precarious employment are rooted in economic and political changes from the late twentieth century onwards [5]. These structural changes fuelled concerns that persistent precarious employment, where workers are exposed to dimensions of precarity over an extended period of time, is increasingly normalized within labour markets and may present a particular problem for health [6–9]; compared to more transitory exposure to precarious employment, which may act as a stepping stone to more secure forms of work [10]. A systematic review found that persistent precarious employment is associated with poorer health, although causality was uncertain [11].

The UK experienced a decade of economic instability following the 2008 recession [12], with increasing unemployment peaking in 2011 and not returning to pre-recession levels until 2015. Real earnings remained below pre-recession levels until 2014 and largely stalled until 2020. Prevalence of precarious employment based on multi-dimensional measures of precarious employment have increased [13,14] or remained constant [15] in a number of high-income countries.

While it is valuable to consider the multi-dimensionality of precarious employment, there is also value in focusing on specific dimensions that may be independently important. Although most are neither necessary nor sufficient characteristics of precarious employment, a better understanding of the relationship between specific dimensions and health can help direct policy. We selected three dimensions that are relatively common in modern labour markets: non-permanent employment contract, employment discontinuity and multiple employment. A non-permanent employee is at greater risk of job loss than a permanent employee and may have fewer contractual protections. Employment discontinuity may also reflect a lack of employee protections. Having more than one job over time (multiple employment) may indicate a single source of employment being insufficient to meet needs.

We anticipated the prevalence of precarious employment to have increased, and that we might expect different patterns of precarious employment between the two time periods of interest. We aimed to describe the prevalence and persistence of dimensions of precarious employment over two time periods (2011–15 and 2015–19), and investigate prevalence of precarious employment patterns, poor self-rated health and common mental disorder.

## METHODS

We hypothesized that the prevalence of these persistently precarious latent classes may vary over time depending on economic conditions, and that they will have a higher prevalence of poor self-rated health and common mental disorder compared to more securely employed latent classes. We undertook descriptive analysis of working-age adults in the United Kingdom Household Longitudinal Study (UKHLS), a representative household panel survey that allows exploration of UK society over time [16], to explore how sequenced employment data might help identify persistently precarious workers not explicitly identified by existing variables. The University of Essex Ethics Committee has approved all data collection on the UKHLS.

We used waves 3-10 of the UKHLS so that the sample focused on two periods of interest. The first includes waves 3 (2011–12) to 6 (2014–15), representing the high-unemployment/low-income period. The second includes waves 7 (2015–16) to 10 (2018–19), representing the lower unemployment/stagnant income period. For the sample to be representative of the working-age UK population, it was important to apply weights from one of the survey waves. We focused on respondents who participated in the final wave of each period (i.e. waves 6 or 10) as this maximized response over the preceding waves. Participants were excluded if they were not aged 20–64 at this final wave. We applied cross-sectional survey weights from waves 6 or 10 to re-weight the sample to resemble the UK working-age population at that time [17]. Note that this excludes respondents with weights of zero due to over-sampling within the survey design (i.e. they do not contribute towards a representative sample). Respondents were also excluded if they had missing data on exposures of interest, outcomes of interest, age or sex. We have reported the number of cases by reason for exclusion in the results section.

We investigated three dimensions of precarious employment measured in every wave of UKHLS as our exposures of interest: employment contract, employment continuity and multiple employment. We classified employment contract status as permanent job, non-permanent job, or non-employed (unemployed or otherwise out of the labour market) at the time of interview. We classified employment spells since the previous survey as continuously employed, discontinuously employed (one or more employment spells plus one or more spells of non-employment), or continuously non-employed. We classified multiple employment as single employment, multiple employment (more than one paid job), or non-employment at the time of interview. Details of how we derived exposure variables are in Table 1 (available as Supplementary data at *Occupational Medicine* Online).

**Table 1.** Weighted analytic sample characteristics at study endpoint (UKHLS waves 6 and 10)

		2011–15: high unemployment/low-income	2015–19: lower unemployment/stagnant income
*N* (weighted)		15 804	12 926
Sex (%)	Male	7616 (48)	6091 (47)
	Female	8187 (52)	6835 (53)
Age (mean (SD))		37 (10)	38 (10)
Employment contract (%)	Non-permanent	779 (5)	804 (6)
	Non-employed	2438 (15)	2023 (16)
	Permanent	12 587 (80)	10 100 (78)
Employment continuity (%)	Discontinuous employment	1493 (9)	1063 (8)
	No employment spells	1703 (11)	1546 (12)
	Continuous employment	12 608 (80)	10 317 (80)
Multiple employment (%)	No	14 621 (93)	12 011 (93)
	Yes	1182 (8)	916 (7)
Self-rated health (%)	Excellent	2918 (19)	1354 (11)
	Very good	5912 (37)	2072 (16)
	Good	4284 (27)	4252 (33)
	Fair	1920 (12)	797 (6)
	Poor	770 (5)	4452 (34)
GHQ-12 (%)	0–3	12 903 (82)	10 202 (79)
	4 or more	2900 (18)	2724 (21)
SF-12 PCS (mean [SD])		51 (10)	50 (10)
SF-12 MCS (mean [SD])		49 (10)	47 (11)

We measured two outcomes of interest at wave 6 (2011–15) and wave 10 (2015–19). Self-rated health is an established measure of general health using a five-point scale [18], which we dichotomised as ‘fair/poor’ or ‘excellent/very good/good’. The 12-item General Health Questionnaire (GHQ 12) is a clinically validated screening tool for common mental disorders [19]. It does not capture other less common mental conditions; however, these are likely to be responsible for a very small number of cases in our sample. We used a score of four or more to indicate caseness (likely common mental disorder).

All analysis was undertaken using R (v4.1.1). We descriptively summarized gender, age, exposure variables and outcome variables at the study endpoint. Categorical variables were presented as numbers and percentages of participants, and continuous variables as mean or median value.

Sequence analysis is a method for analysing longitudinal data where individuals have different states over time. We used sequence analysis to create an employment history dataset for each dimension using the TraMineR package [20], where the employment state is recorded for each timepoint. Latent class analysis (LCA) is a method for identifying groups (classes) that have shared (but unmeasured) characteristics within a population, by using other variables included in the data.

After sequence analysis, we used LCA to generate parsimonious summary measures of exposure to persistent precarity from each of our sequenced datasets using the PoLCA package [21]. We ran seven latent class models for each dimension, specifying between two and eight latent classes. We selected an optimal model for each dimension, based on inspection of Bayesian Information Criterion scores and assessment of face validity. The optimal model formed the basis for a new employment history categorical variable with the latent classes providing the values. We presented employment histories as sequence frequency plots grouped by latent class membership. Normalized entropy scores were calculated for the LCA models to assess accuracy of classification.

After establishing latent class measures of precarity persistent, we then calculated age-sex standardised prevalence of poor self-rated health and common mental disorder by latent class for each dimension using the 2013 European Standard Population (ESP 2013) and the Epitools package in R [22]. The ESP is a theoretical population adding up to a total of 100 000 based on the age-sex distribution of Europe in 2013. It allows us to calculate rates that are comparable across populations with differing age-sex structures. Because the accuracy of direct standardised rates is affected by the number of cases within specific strata, we undertook, as a sensitivity analysis, a series of logistic regression models with poor self-rated health or common mental disorder as the outcome, and latent classes as the exposures. Logistic regression is potentially more statistically efficient and less prone to sparse data biases than direct standardisation. Our regression models are presented in the Supplementary Materials (available as Supplementary data at *Occupational Medicine* Online). For each set of exposure classes, the class most closely representing stable employment was the reference category. All models controlled for sex and age group to provide similar adjustment to our standardized rates for basic demographic factors by which the distribution of exposures and outcomes would reasonably be expected to vary. We have not controlled for further covariates as the purpose of this study is to describe health needs rather than to provide causal estimates [23,24].

## RESULTS

**Figure 1.**
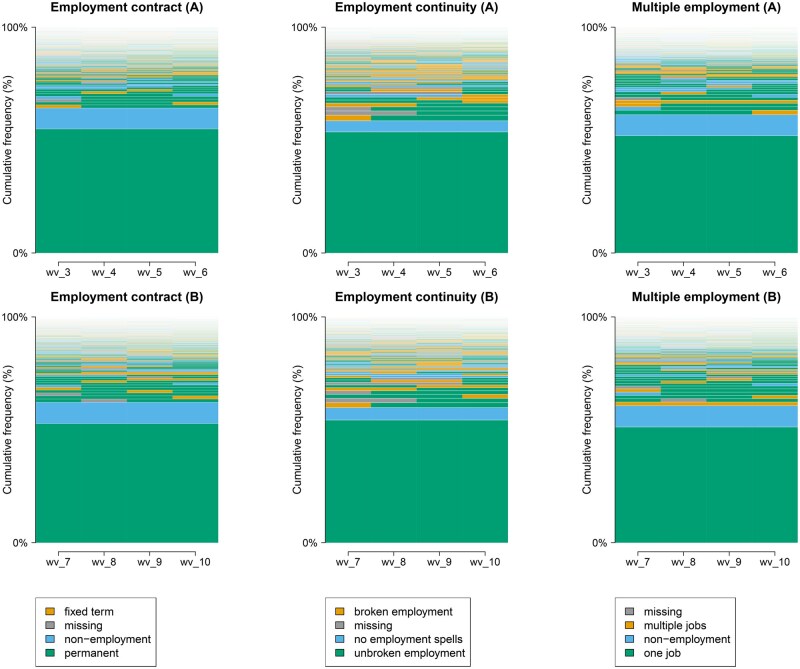
Employment history exposure sequences ascending in order of frequency; employment history sequences are plotted horizontally along the x-axis, with each block representing a survey wave and sorted vertically along the y-axis in order of frequency.

**Figure 2.**
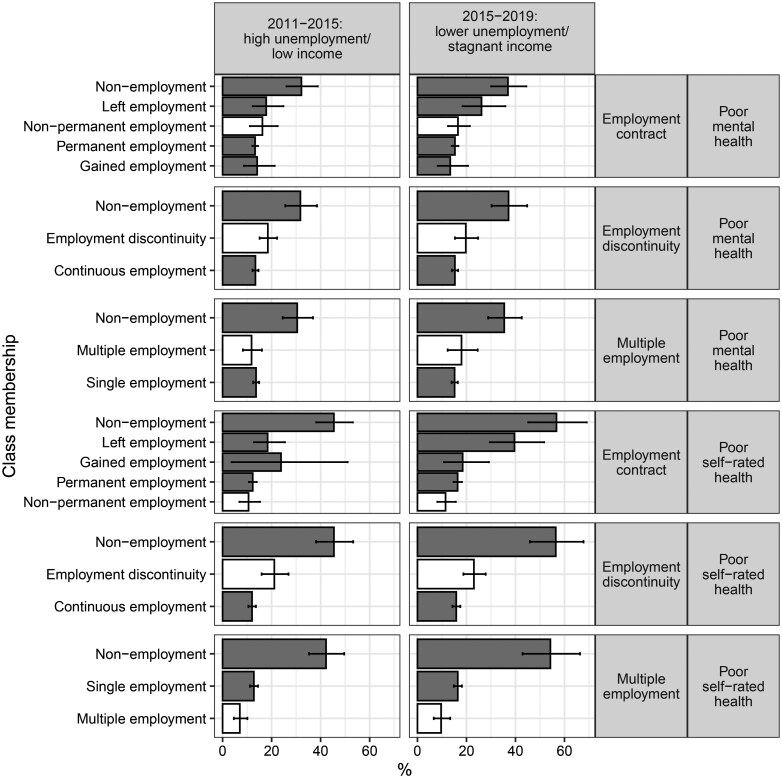
Age-sex standardised prevalence of mental health and poor self-rated health by precarious employment dimension and latent class membership

A total of 45 188 individuals participated in UKHLS waves 3-6 and 34 318 in waves 7–10. We excluded 13 258 and 9 751 individuals, respectively, for not being aged 20–64 at study endpoint; 8375 and 4835 individuals with zero weights; 5938 and 5175 retired individuals; and 1456 and 1594 individuals who reported missing data for key exposure, outcome or covariate variables at study endpoint. This left unweighted analytic samples of 16 161 and 12 963 individuals in 2011–15 and 2015–19; and weighted samples of 15 804 and 12 926. Table 1 presents key characteristics of the analytic sample at the study endpoint. Responses to all four waves in each study period were provided by 97% of the analytic sample for 2011–15 and 96% for 2015–19 (Figure 1, available as Supplementary data at *Occupational Medicine* Online). Valid response sequences are presented to demonstrate wave missingness in Figure 2 (available as Supplementary data at *Occupational Medicine* Online).

For each exposure dimension, consistent secure employment—permanent employment, continuous employment or single employment—was the most observed sequence, followed by consistent non-employment across both samples (Figure 1). A large minority of cases had employment sequences with varying states during the study periods, including exposure to the dimensions of precarious employment.

We undertook LCA to better understand these less-common sequences. LCA of employment contract history identified a five-class model as the optimum solution for both periods. We described these classes as individuals: in permanent employment for most or all the period (permanent class); non-employed for most or all of the period (non-employment class); who typically moved into employment during the period (gained employment class); who typically moved out of employment during the period (left employment class); or typically in non-permanent employment during the period (non-permanent class) (Table 2, Figures 3–5, available as Supplementary data at *Occupational Medicine* Online). The non-permanent employment class represented 5% of our sample in both 2011–15 and 6% in 2015–19.

**Table 2.** Weighted overlap of persistent precarious employment latent class overlap at study endpoint (UKHLS waves 6 and 10)

	2011–15		2015–19	
	*N*	%	*n*	%
No membership of precarious employment classes	12 543	79	10 178	79
Non-permanent contract class only	429	3	507	4
Employment discontinuity class only	1602	10	1218	9
Multiple employment class only	869	6	744	6
Non-permanent contract and employment discontinuity classes	206	1	130	1
Non-permanent contract and multiple employment classes	81	0.5	81	0.6
Employment discontinuity and multiple employment classes	60	0.4	55	0.4
Membership of all three precarious employment classes	15	0.1	14	0.1

LCA of employment discontinuity identified a three-class model as the optimum solution for both periods. We described these classes as individuals: typically in continuous employment for most or all the period (continuous employment class); typically non-employed for most or all the period (non-employment class); or who typically reported employment discontinuity for most or all waves (employment discontinuity class) (Table 3, Figures 6–8, available as Supplementary data at *Occupational Medicine* Online. The employment discontinuity class represented 12% of our sample in 2011–15 and 11% in 2015–19.

LCA of multiple employment identified a three-class model as the optimum solution for both periods. We described these classes as individuals: typically in single employment for most or all the period (single employment class); typically non-employed for most or all of the period (non-employment class); and typically in multiple employment during the period (multiple employment class) (Table 4 and Figures 9–11, available as Supplementary data at *Occupational Medicine* Online). The multiple employment class represented 7% of our sample in both 2011–15 and 2015–19.

The normalized entropy score for each optimal latent class model was greater than 0.9, suggesting a high level of accuracy of class assignment (Table 4, available as Supplementary data at *Occupational Medicine* Online). We found little overlap between the latent classes representing persistent exposure to the three dimensions (Table 2). Only 0.1% in both 2011–15 and 2015–19 were members of all three persistent precarious employment classes.

Age-sex standardized prevalence of poor self-rated health increased for all employment classes between our two study periods, except for the gained employment class within the employment contract dimension (Figure 2). Persistent non-employment classes had the highest prevalence in all three dimensions. Prevalence of poor self-rated health among the non-permanent employment contract class (11%) was similar to the permanent employment class (12%) in 2011–15. However, by 2015–19, the permanent employment class’s prevalence (16%) was higher than that for the non-permanent class (11%). Prevalence of poor self-rated health for the employment discontinuity class was 21% in 2011–15 and 23% in 2015–19, compared with 12% and 16% among the continuous employment class. Prevalence among the persistent multiple employment class was lower in both samples (7% in 2011–15 and 10% in 2015–19) compared with the single employment class (13% in 2011–15 and 16% in 2015–19).

Similarly, prevalence of common mental disorders increased for all employment classes between the two periods and was highest among the non-employment classes for all three dimensions (Figure 2). Age-sex standardized prevalence of common mental disorder among the non-permanent employment class rose from 16% in 2011–15 to 17% in 2015–19, but at a lower rate of increase than for the permanent employment class (13–15%). Prevalence among the employment discontinuity class was 19% in 2011–15 and 20% in 2015–19, compared to 13% and 14% for the continuous employment class. Prevalence among the multiple employment class (12%) was lower than the single employment class (14%) in 2011–15. However, the multiple employment class’s prevalence rose to 18% in 2015–19 compared to 15% in the single employment class.

## DISCUSSION

We found that patterning and prevalence of dimensions of persistent precarious employment and associated health needs in the UK remained stable despite substantial variation in the political and macroeconomic context across two distinct periods—high unemployment and low-income (2011–15); and relatively lower unemployment and stagnant income (2015–19).

We identified three persistent precarious employment latent classes, one representing each of the dimensions that we investigated as exposures of interest (non-permanent employment contract, employment discontinuity and multiple employment). The latent class analyses did not identify other classifications that we had theorized may be present before analysis (e.g. classes representing participants moving in or out of precarious employment) based on previous studies that have used similar methods [8,9,25]. Similar patterns were observed across the three dimensions of persistent precarious employment, with sequences in each precarious employment class typically featuring the precarious employment statein two or more waves.

Despite similarities between these three persistent precarious employment dimensions, there was little crossover between these latent classes, suggesting that these are distinct groupings of precariously employed workers. Researchers and policymakers should therefore be careful not to interpret a single dimension of precarious employment as representing the multidimensional concept.

We found that the prevalence of self-rated health and common mental disorder increased for almost all latent classes between 2011–15 and 2015–19. Reinforcing the distinct nature of these dimensions, we found that the prevalence of self-rated health and common mental disorder for the persistent precarious employment classes varied by exposure dimension. The employment discontinuity class had the highest prevalence of health harms of the three persistent precarious employment classes. Similarly, a study of employment trajectories using the Swiss Household Panel study found discontinuous employment histories were associated with lower self-rated health and mental health outcomes [26].

We found lower-than-expected prevalence of common mental disorder based on a previous meta-analysis of studies reporting an association between persistent non-permanent employment and poor mental health [11]. Previous studies that investigated the association between persistent non-permanent employment and self-rated health reported mixed findings [27–29].

Previous evidence on multiple employment and health outcomes has also been inconclusive, finding no association [30] or better health outcomes [31]. However, there is evidence to suggest that health harms may be more likely among workers with more than two jobs [32]. One possible reason for the apparent lack of association between having multiple jobs and health outcomes may be a healthy worker effect phenomenon [33], where the ability to hold multiple jobs is dependent on better health status, regardless of the effect of having multiple jobs.

We found that the employment discontinuity latent class was the most prevalent of the three persistent precarious employment dimensions. It also had the most consistently poorer health outcomes. On this basis, and the existing evidence base regarding non-employment and health [34,35], employment discontinuity would appear to be an appropriate exposure to target from a public health perspective.

The other two dimensions of precarious employment (non-­permanent employment and multiple employment classes) were less prevalent and inconsistently associated with our outcomes of interest. This suggests that they would be lower priorities in terms of targeted public health intervention. However, further research using robust quantitative methods is required to better understand the extent to which the relationship between precarious employment and health outcomes are causal. In addition, there remains a gap in effectiveness evidence for interventions seeking to reduce exposure to precarious employment [36]. Further research would also be valuable to better understand how multiple employment impacts on health and other outcomes, especially among those on lower incomes.

There are several limitations that should be considered when interpreting the findings of our study. First, our study design was intended to provide exploratory and descriptive evidence and should not be interpreted as representing causal relationships. Second, we have not accounted for missing data due to attrition or item non-response, which may introduce bias to our findings. We limited to valid cases at study endpoint but used complex sample weights to estimate a sample representative of the general UK population. For sequence analysis, we treated item non-response as a sequence state as few cases had one or more missing data points. Third, our exposures represent dimensions of precarious employment but do not necessarily indicate its presence on their own, nor do they capture the totality of precarious employment as a multi-dimensional concept. However, the limited cross-over between the three dimensions suggests value in looking at dimension-specific exposures to precarious employment. This is particularly true when considering potential policies to reduce exposure to precarious employment. Fourth, the R package we identified as the best option for undertaking LCA was not able to apply complex survey weights. However, both the preceding sequence analysis and subsequent analysis of outcomes by latent class were undertaken using complex survey weights, which we believe limits any bias arising from the LCA stage of our analysis.

Our findings indicate a minority of workers in the UK who were in a state of chronic precarity, which did not change substantially between the two periods we investigated. The distinct persistent precarious employment classes identified within the data indicate that while dimension-specific policy interventions are necessary, they are unlikely to be sufficient in terms of addressing the multi-dimensional nature of precarious employment. Of the dimensions of precarious employment that we investigated, addressing employment discontinuity appears most promising in terms of reducing health harms.

## Supplementary Material

kqaf064_Supplementary_Data

## Data Availability

The data underlying this article are available in University of Essex, Institute for Social and Economic Research. (2021). Understanding Society: Waves 1-10, 2009-2019 and Harmonised BHPS: Waves 1-18, 1991-2009. [data collection]. 13th Edition. UK Data Service. SN: 6614, at http://doi.org/10.5255/UKDA-SN-6614-14.
