# Supplementary material for: Prevalence of poor self-rated health and common mental disorder among persistently precarious employed adults
Source: Occup Med (Lond). 2025 Sep 22;75(6):369–76. doi: 10.1093/occmed/kqaf064 (PMC12449262; doi:10.1093/occmed/kqaf064)
Supplement: kqaf064_Supplementary_Data [file kqaf064_supplementary_data.docx]

# Supplementary materials

## User defined variable definitions

| **Name** | **Description** |
| --- | --- |
| Employment contract | Respondent is has (a) permanent job or (b) a job which is in some way not permanent. A derived variable was created for analytic purpose using this variable and current labour force status to identify participants who were unemployed or not in employment. |
| Employment continuity | Respondent has been (a) continuously employed, (b) discontinuously employed (one or more employment spell plus one or more spell of non-employment or unemployment), or (c) continuously non-employed since previous wave (unemployed of non-employed/inactive. Derived variable from number of employment spells since last interview, number of non-employment spells since last interview and number of unemployment spells since last interview. |
| Multiple employment | Respondent has more than one paid job at time of interview. A derived variable was created for analytic purpose using this variable and current labour force status to identify participants who were unemployed or not in employment. |

**Supplementary table 1: User derived variables**

## Missing data (unweighted)


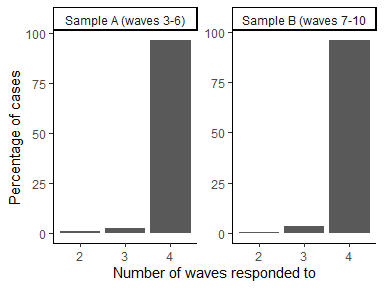


**Supplementary figure 1: Number of waves responded to by percentage of the analytic sample (unweighted)**

## Sequence analysis of precarious employment exposures of interest


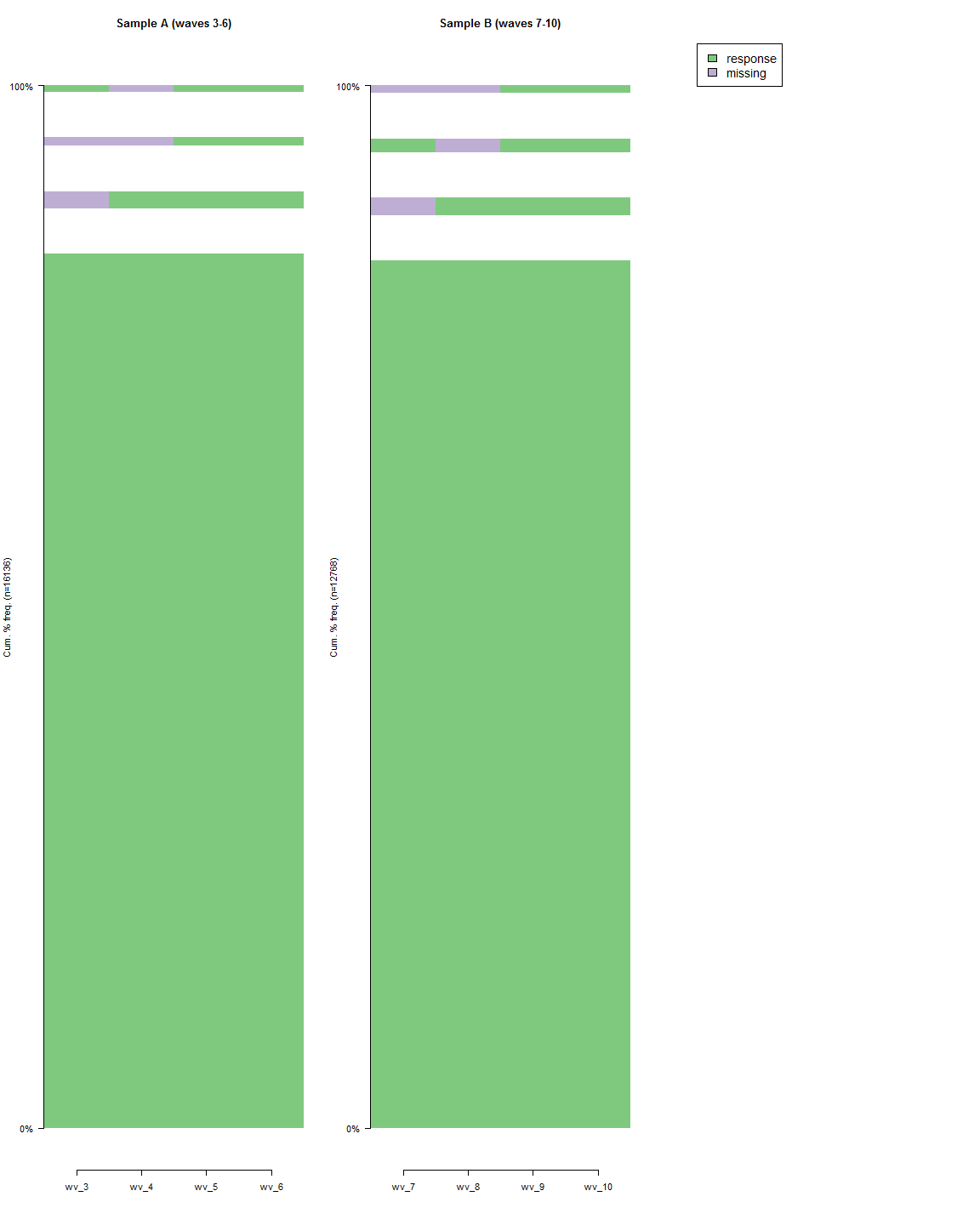


**Supplementary figure 2: Valid response sequences ascending in order of frequency (unweighted) response state (response or missing) sequences are plotted horizontally along the x-axis with each block representing a survey wave and sorted vertically along the y-axis in order of frequency.**

## Latent class analysis - Non-permanent employment

|  | 2011-2015 | | | 2015-2019 | | |
| --- | --- | --- | --- | --- | --- | --- |
| Number of classes | BIC | AIC | Chi^2^ | BIC | AIC | Chi^2^ |
| 2 | 54153 | 54022 | 53906 | 44090 | 43963 | 67193 |
| 3 | 50857 | 50657 | 1435 | 40593 | 40399 | 1681 |
| 4 | 50508 | 50239 | 652 | 40223 | 39962 | 523 |
| 5 | 50177 | 49838 | 179 | 40017 | 39689 | 206 |
| 6 | 50209 | 49801 | 117 | 40026 | 39631 | 123 |
| 7 | 50229 | 49752 | 51 | 40046 | 39583 | 66 |
| 8 | 50314 | 49768 | 48 | 40125 | 39594 | 55 |

**Supplementary table 2: Model fit statistics for non-permanent employment contract latent class analysis solutions (2-8 classes) - 2011-2015 and 2015-2019**


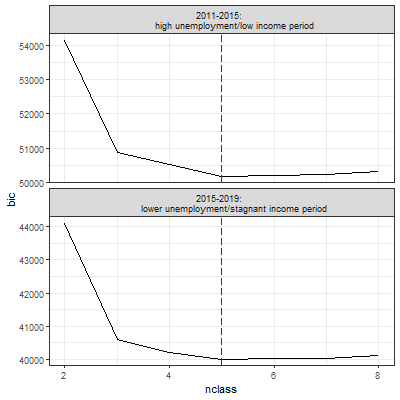


**Supplementary figure 3: BIC elbow plot for non-permanent employment contract latent class analysis solutions (2-8 classes) - 2011-2015 and 2015-2019**


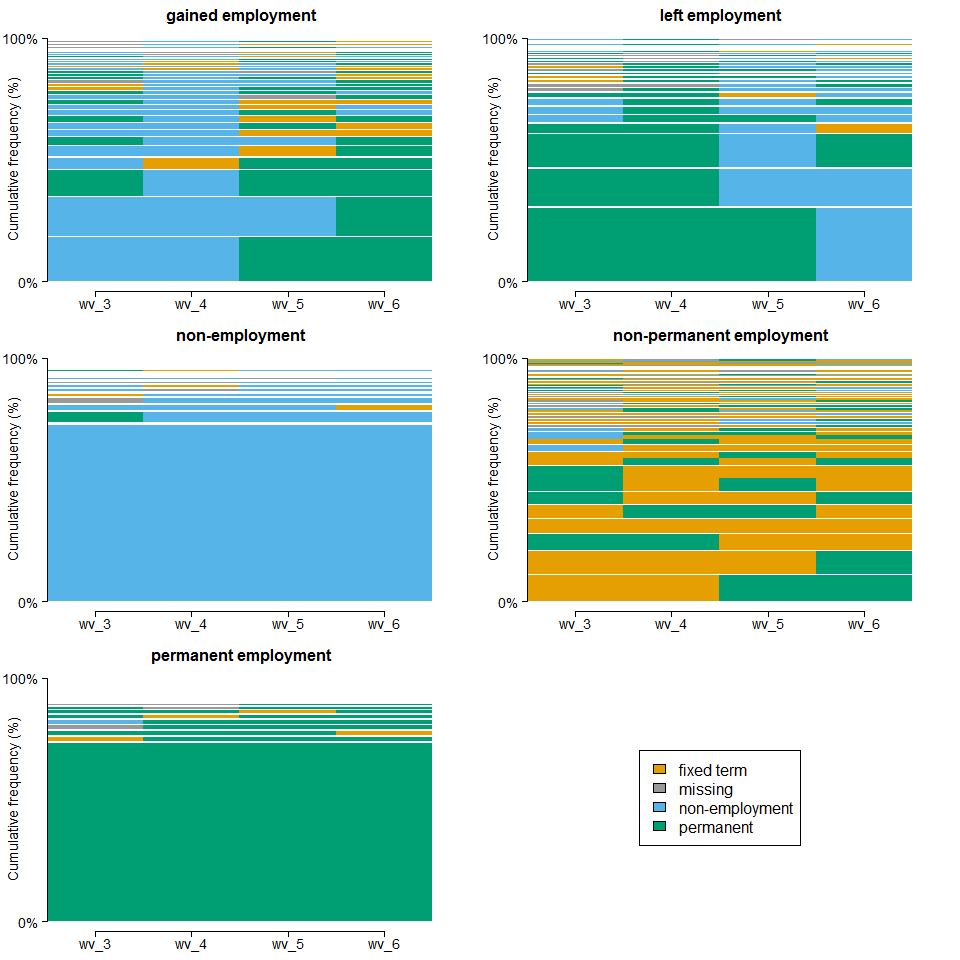


**Supplementary figure 4: Employment contract sequences ascending in order of frequency grouped by latent class membership - 2011-2015; employment history sequences are plotted horizontally along the x-axis with each block representing a survey wave and sorted vertically along the y-axis in order of frequency.**


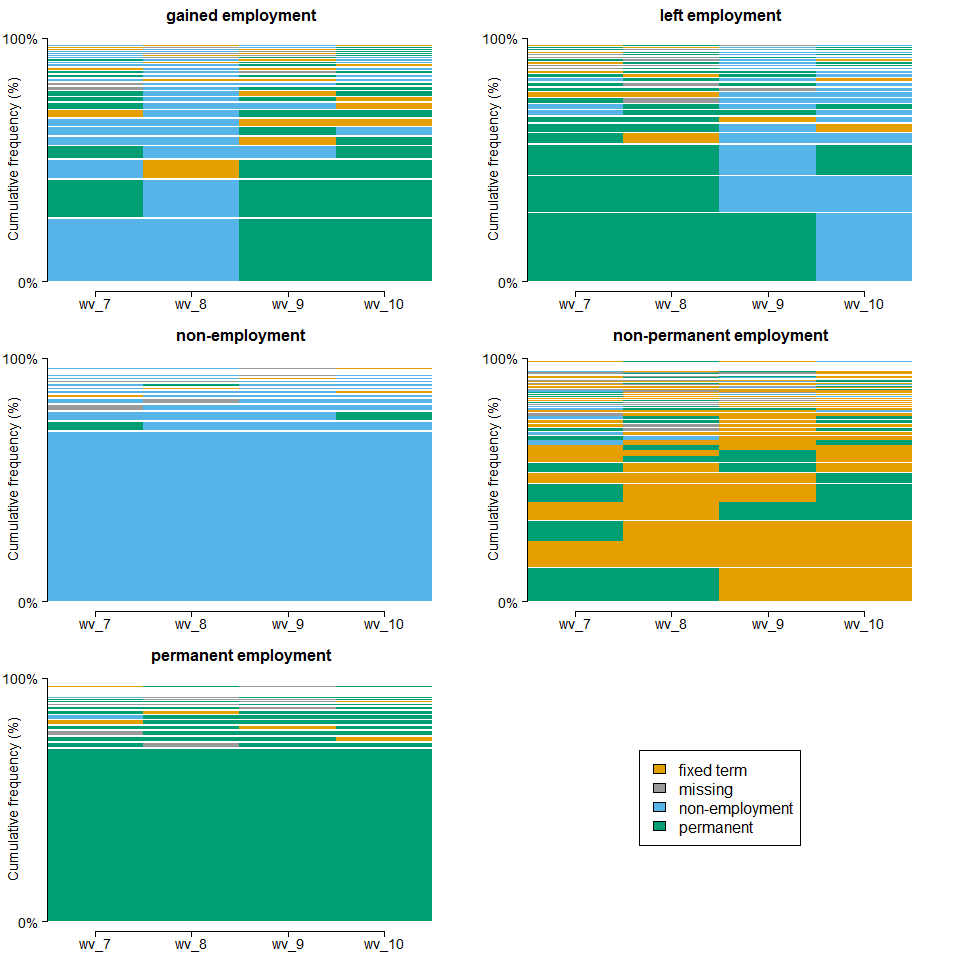


**Supplementary figure 5: Employment contract sequences ascending in order of frequency grouped by latent class membership - 2015-2019; employment history sequences are plotted horizontally along the x-axis with each block representing a survey wave and sorted vertically along the y-axis in order of frequency.**

## Latent class analysis - Employment discontinuity

### Employment discontinuity - 2011-2015

|  | 2011-2015 | | | 2015-2019 | | |
| --- | --- | --- | --- | --- | --- | --- |
| Number of classes | BIC | AIC | Chi^2^ | BIC | AIC | Chi^2^ |
| 2 | 56679 | 56548 | 11346 | 42968 | 42841 | 8506 |
| 3 | 52264 | 52064 | 3161 | 39653 | 39458 | 2105 |
| 4 | 51311 | 51042 | 1696 | 38924 | 38663 | 909 |
| 5 | 51427 | 51088 | 1762 | 39084 | 38756 | 1099 |
| 6 | 50713 | 50306 | 738 | 38842 | 38446 | 642 |
| 7 | 51019 | 50542 | 969 | 38895 | 38432 | 642 |
| 8 | 50223 | 49677 | 73 | 38868 | 38338 | 511 |

**Supplementary table 3: Model fit statistics for employment continuity latent class analysis solutions (2-8 classes) - 2011-2015 and 2015-2019**


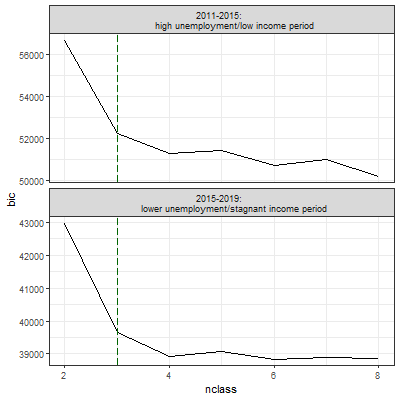


**Supplementary figure 6: BIC elbow plot for employment continuity latent class analysis solutions (2-8 classes) - 2011-2015 and 2015-19**


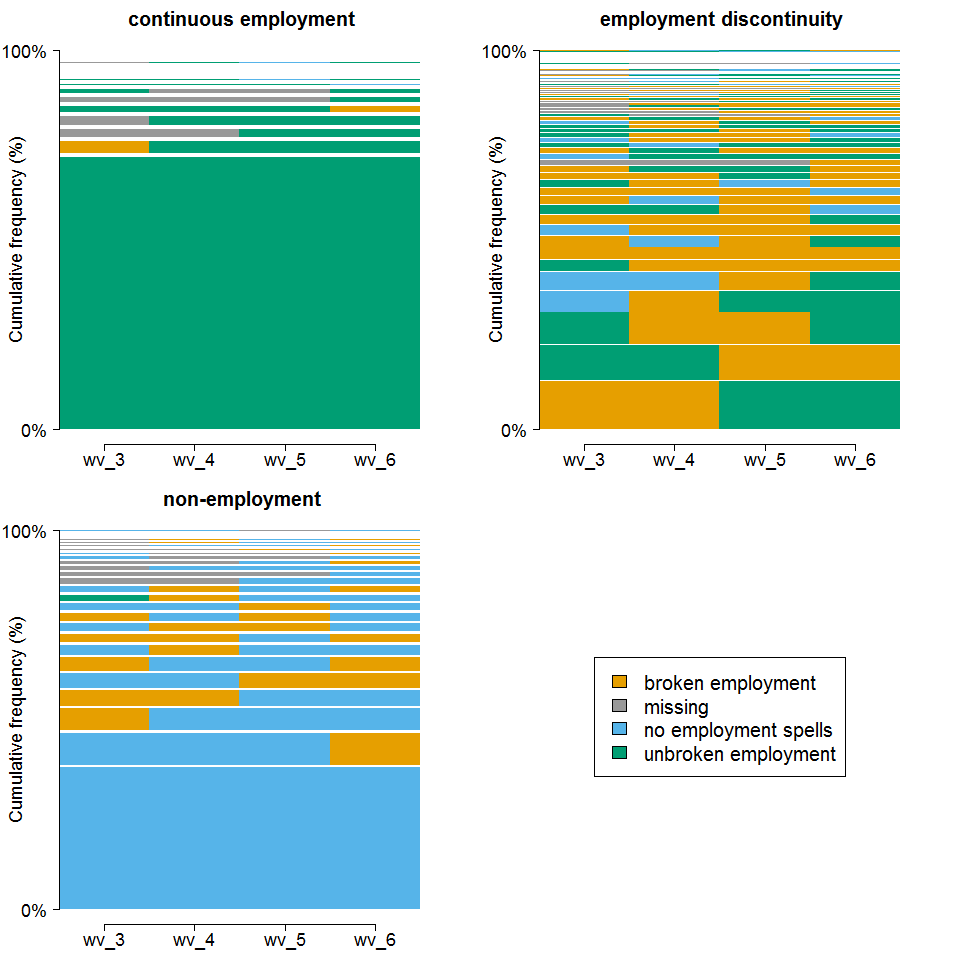


**Supplementary figure 7: Employment continuity sequences ascending in order of frequency grouped by latent class membership - 2011-2015; employment history sequences are plotted horizontally along the x-axis with each block representing a survey wave and sorted vertically along the y-axis in order of frequency.**


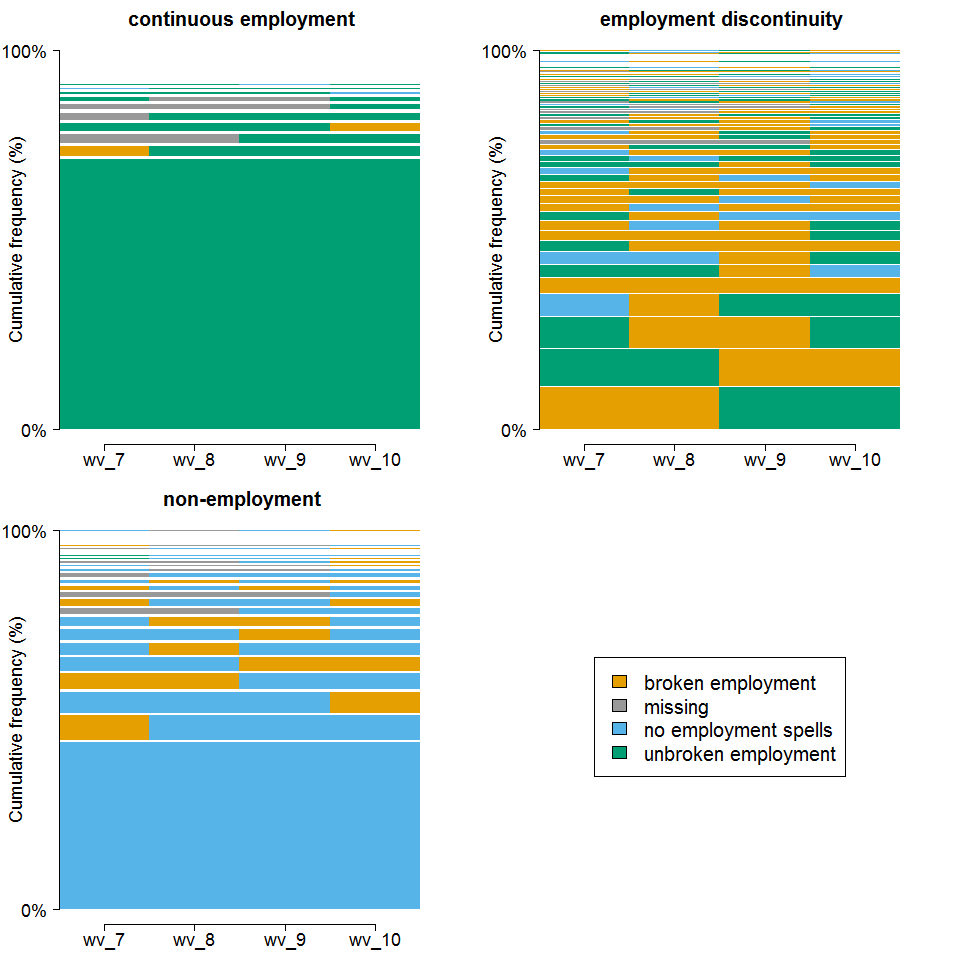


**Supplementary figure 8: Employment continuity sequences ascending in order of frequency grouped by latent class membership - 2015-2019; employment history sequences are plotted horizontally along the x-axis with each block representing a survey wave and sorted vertically along the y-axis in order of frequency.**

## Latent class analysis - Multiple employment

### Multiple employment - 2011-2015

|  | 2011-2015 | | | 2015-2019 | | |  |
| --- | --- | --- | --- | --- | --- | --- | --- |
| Number of classes | BIC | AIC | Chi^2^ | BIC | AIC | Chi^2^ |  |
| 2 | 60268 | 60137 | 144017 | 47113 | 46986 | 156961 |  |
| 3 | 54671 | 54471 | 1699 | 41712 | 41518 | 1542 |  |
| 4 | 54035 | 53766 | 519 | 41256 | 40994 | 448 |  |
| 5 | 53825 | 53487 | 174 | 41261 | 40932 | 344 |  |
| 6 | 53822 | 53415 | 92 | 41246 | 40850 | 244 |  |
| 7 | 53904 | 53427 | 88 | 41207 | 40744 | 120 |  |
| 8 | 53939 | 53393 | 25 | 41191 | 40661 | 21 |  |

**Supplementary table 4: Model fit statistics for multiple employment latent class analysis solutions (2-8 classes) - 2011-2015 and 2015-2019**


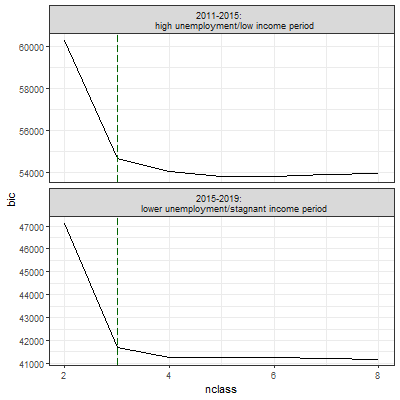


**Supplementary figure 9: BIC elbow plot for multiple employment latent class analysis solutions (2-8 classes) – 2011-2015 and 2015-2019**


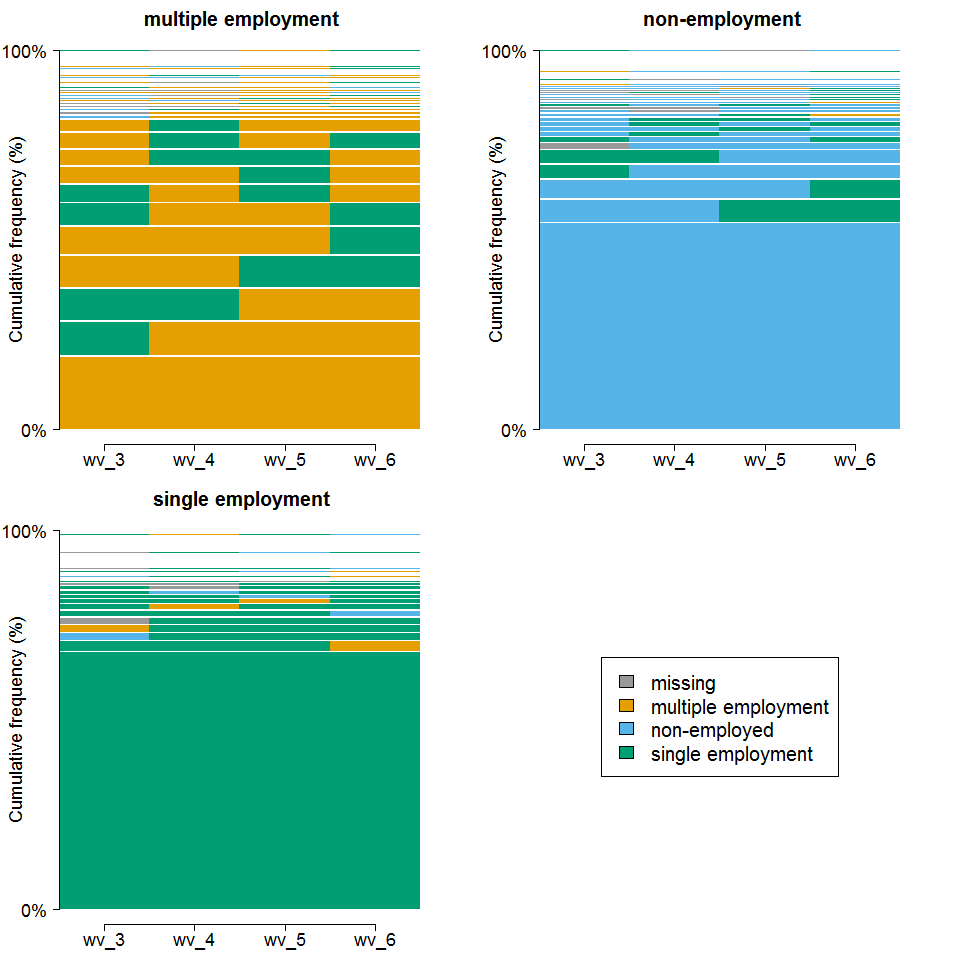


**Supplementary figure 10: Multiple employment sequences ascending in order of frequency grouped by latent class membership - 2011-2015; employment history sequences are plotted horizontally along the x-axis with each block representing a survey wave and sorted vertically along the y-axis in order of frequency.**


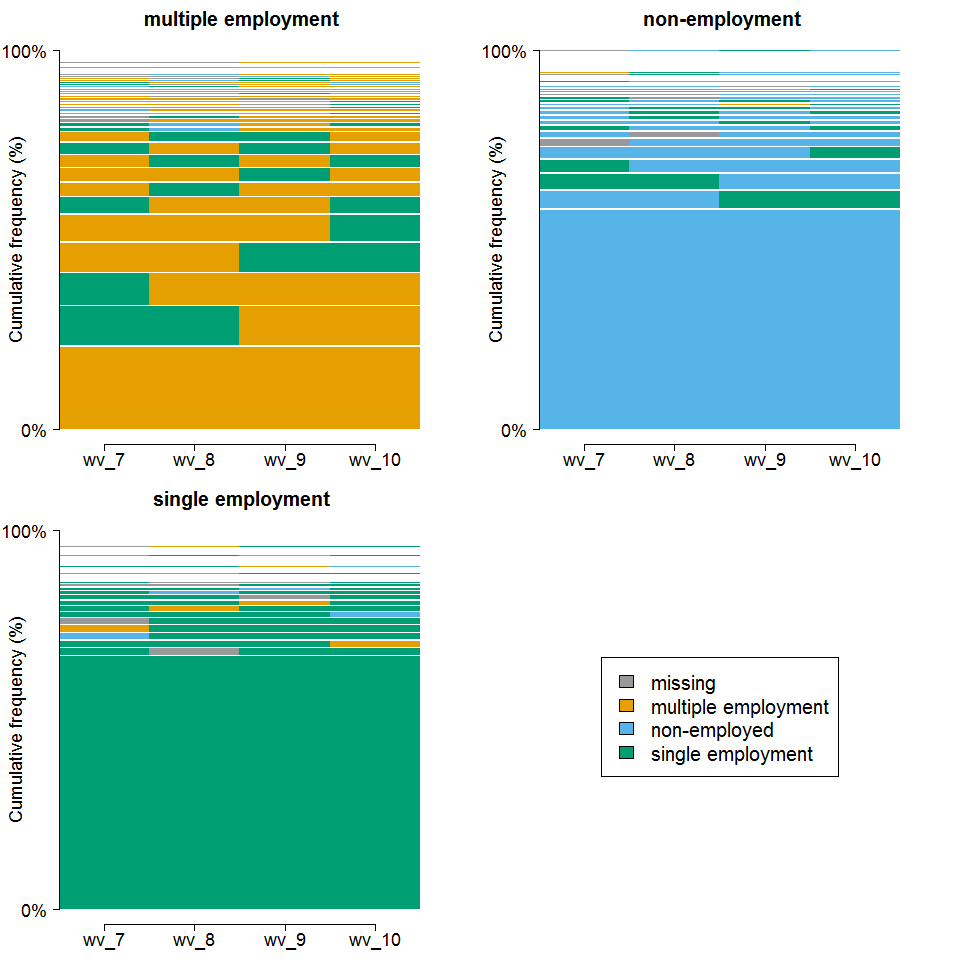


**Supplementary figure 11: Multiple employment sequences ascending in order of frequency grouped by latent class membership - 2015-2019; employment history sequences are plotted horizontally along the x-axis with each block representing a survey wave and sorted vertically along the y-axis in order of frequency.**

## Latent class analysis – optimal model entropy scores

| **Latent class model** | **Entropy** |
| --- | --- |
| Employment contract (2011-2015) | 0.94 |
| Employment contract (2015-2019) | 0.94 |
| Employment continuity (2011-2015) | 0.93 |
| Employment continuity (2015-2019) | 0.93 |
| Multiple employment (2011-2015) | 0.94 |
| Multiple employment (2015-2019) | 0.95 |

**Supplementary table 5: Normalised entropy scores for optimal latent class analysis models**

# Regression analysis

## Self-rated health

The odds for poor self-rated health were found to be higher among members of the employment discontinuity class (OR 2.41, 95%CI 2.07 to 2.81 in 2011-2015; OR 2.20, 95%CI 1.85 to 2.61 in 2015-2019) compared to the continuous employment class (Supplementary figure 12); while the odds of poor self-rated health were lower for the persistent multiple employment class compared to the persistent single employment class (OR 0.69, 95%CI 0.54 to 0.89 in 2011-2015; OR 0.66, 95%CI 0.51 to 0.86 in 2015-2019). The odds of poor self-rated health were not found to differ for members of the non-permanent class (OR 1.19, 95%CI 0.92 to 1.54 in 2011-2015; OR 0.92, 95%CI 0.67 to 1.26 in 2015-2019) compared to the permanent employment class. We found consistent evidence across the three precarious employment dimensions that persistent non-employment was associated with higher odds of poor self-rated health than persistent employment. In our employment contract model gaining or leaving employment were also associated with higher odds of poor self-rated health.


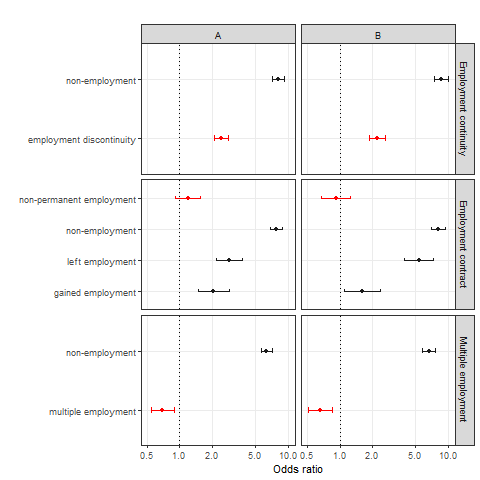


**Supplementary figure 12: The odds of poor self-rated health by precarious employment history class compared to more securely employed reference group (2011-2015 and 2015-2019)**

Note: Reference categories: permanent employment class (non-permanent employment contract), continuous employment class (employment discontinuity), single employment class (multiple employment). Persistently precarious employment class is marked as red. All models adjusted for age group and gender.

## Common mental disorder

We found consistent evidence across the three precarious employment dimensions that being persistently non-employed or, in the case of the employment contract model - leaving employment was associated with higher odds of poor self-rated health and common mental disorder. In contrast, membership of persistent precarious employment classes was inconsistently associated with common mental disorders (Supplementary figure 13). The non-permanent employment class was not clearly associated with higher odds of GHQ-12 caseness compared to the permanent employment class in 2011-2015 (1.23 OR, 95%CI 0.97 to 1.57) or 2015-2019 (1.22 OR, 95%CI 0.95 to 1.57). The odds of GHQ-12 caseness were higher among members of the employment discontinuity class compared to the continuous employment class in 2011-2015 (1.56 OR, 95%CI 1.36 to 1.80) and in 2015-2019 (1.37 OR, 95%CI 1.17 to 1.62). Membership of the multiple employment class was not clearly associated with GHQ-12 caseness in 2011-2015 (0.90 OR, 95%CI 0.74 to 1.09) or in 2015-2019 (1.16 OR, 95%CI 0.94 to 1.43) compared to the single employment class.


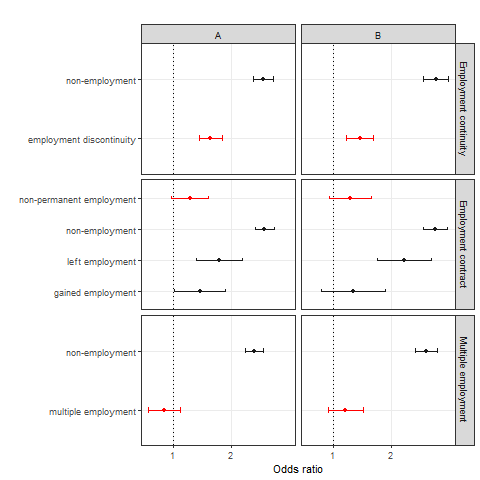


**Supplementary figure 13: The odds of common mental disorder by precarious employment history class compared to more securely employed reference group for study periods 2011-2015 (high unemployment/low-income) and 2015-2019 (lower unemployment/stagnant income)**

Note: Reference categories: permanent employment class (non-permanent employment contract), continuous employment class (employment discontinuity), single employment class (multiple employment). Persistently precarious employment class is marked as red. All models adjusted for age group and gender.
